# Data of first *de-novo* transcriptome assembly of a non-model species, hawksbill sea turtle, *Eretmochelys imbricate*, nesting of the Colombian Caribean

**DOI:** 10.1016/j.dib.2017.10.015

**Published:** 2017-10-11

**Authors:** Javier Hernández-Fernández

**Affiliations:** Genetics, Molecular Biology and Bioinformatics Lab, Department of Natural and Environmental Sciences, Universidad Jorge Tadeo Lozano, Cra. 4 N° 22-61 modulo 7 Piso 6, Bogotá D.C., Colombia

**Keywords:** Hawksbill turtle, Trinity, RNAseq, illumina, N50

## Abstract

The hawksbill sea turtle, *Eretmochelys imbricata,* is an endangered species of the Caribbean Colombian coast due to anthropic and natural factors that have decreased their population levels. Little is known about the genes that are involved in their immune system, sex determination, aging and others important functions. The data generated represents RNA sequencing and the first de-novo assembly of transcripts expressed in the blood of the hawksbill sea turtle. The raw FASTQ files were deposited in the NCBI SRA database with accession number SRX2653641. A total of 5.7 Gb raw sequence data were obtained, corresponding to 47,555,108 raw reads. Trinity was used to perform a first de-novo assembly, and we were able to identify 47,586 transcripts of the female hawksbill turtle transcriptome with an N50 of 1100 bp. The obtained transcriptome data will be useful for further studies of the physiology, biochemistry and evolution in this species.

**Specifications Table**

**Table:** 

**Subject area**	Genetics and Transcriptomics
**More specific subject area**	Transcriptomics of sea turtles *Eretmochelys imbricata*
**Type of data**	Raw reads of DNA sequences
**How data was acquired**	A blood sample of a living specimen of the sea turtle *Eretmochelys imbricata* was collected for total RNA isolations. Prepared a paired-end library, sequenced by the Hiseq. 2000 system. The obtained data was subjected to de novo assembly.
**Data format**	Raw data FASTQ file
**Experimental factors**	The hawksbill turtle is in captivity in swimming pools with sea water. The concentration of oxygen is 5 mg/L, temperature of 30 °C and salinity of 30.5
**Experimental features**	The de novo assembling of the transcriptome and the functional identification of the genes expressed by hawsbill turtle was performed.
**Data source location**	Islas del Rosario, Bolívar, Colombia 10°17’67.95” N – 75°77’16.27” W
**Data accessibility**	The raw FASTQ files were deposited in the NCBI SRA database with accession number SRX2653641 (https://www.ncbi.nlm.nih.gov/sra/SRX2653641/)

**Value of the data**

• This is the first de novo transcriptome of *E. imbricate* sea turtle published
• The obtained transcriptome data will be useful for further studies of the physiology, biochemistry evolution and others of *E. imbricate* sea turtle.
• It is possible to know and analyze the metabolic pathways in which the genes identified are involved.

## 1 Data

The hawksbill turtle, *Eretmochelys imbricata* [1], is a non-model species that is found throughout the tropics in the central Atlantic and Indo-Pacific regions [2], [3], [4], [5]. Marcovaldi et al. [6] reported that this species nests from the state of Florida (USA) in the wider Caribbean, to the south coast of Espirito Santo in Brazil. Trujillo-Arias et al. [7] located foraging areas for the hawksbill turtle in Colombia on the Islas del Rosario and in the National Natural Park of Cabo de la Vela.

This turtle is listed by the Union for Conservation of Nature as critically endangered A2bd [8] and in Appendix I of CITES [9]. The main causes of the population decline include meat and shells marketing, egg consumption and oil production [10], [11], bycatch in industrial and artisanal fisheries and habitat loss [12], [13]. The demand for shells continues today on the black market [3] with the dramatically decreasing their population [4].

The data of this article are represented by the raw FASTQ files deposited in the NCBI SRA database with accession number SRX2653641 (https://www.ncbi.nlm.nih.gov/sra/SRX2653641/).

The transcriptome sequencing and read processing are summarized in Table 1. We obtained a total of 47,586 assembled transcripts with a N50=1100 bp, average length of 724 bp.

**Table 1:** Statistics of *Eretmochelys imbricata* transcriptome assembly.

No of raw data bases	4,803,065,908
No of reads of pairs	47,555,108
No of assembled transcrips	47,586
Assembly GC percent	46,2
Contig N50	1100
Contig minimum	147
Contig maximum	8,666
Averag contig	724

## 2 Experimental design, materials and methods

### 2.1 Animal materials

A blood sample from a *Eretmochelys imbricata* individual was obtained from the CEINER Oceanarium in San Martin de Pajares Island, Cartagena. The blood was obtained from the dorsal cervical breasts in accordance with Dutton [14] methodology. The sample was placed in sterilized tubes with Tris-EDTA buffer 0.1 M (GreinerBio-one®, Kremsmünster, Austria) solution and was transported at 4 °C to the Molecular Biology laboratory of the Universidad Jorge Tadeo Lozano, Bogota campus. The sample was collected following the ethical standards established by the legislation and the study obtained permission from the Ministry of the Environment for the development of the Biodiversity research (No 24 of June 22, 2012) and the Genetic Resources Access contract (No 64 of April 2013).

A blood sample of a *Eretmochelys imbricata* sea turtle was used for total RNA extraction using the RNeasy Mini Kit (Quiagen, Hilden, Germany) according to the manufacturer's protocol. RNA integrity was confirmed using a 2100 Bioanalyzer (Agilent Technologies). For mRNA library preparation, we used a TruSeq RNA Library Prep Kit v2 according to manufacturer's instructions (Illumina, San Diego, U.S.A.). The quality control of generated libraries was done using the 2100 bio-analyzer (Agilent, Santa Clara, U.S.A.). The library was paired-end sequenced by Macrogen Co. (Seoul, South Korea) using the Hiseq. 2000 Platform. The quality of cleaned raw reads was verified with the FastQC program (http://www.bioinformatics.bbrc.ac.uk/projects/fastqc/). FastQC delivered quality metrics that were used to identify if the data required initial pre-processing before the transcriptome assembly.

### 2.2 De novo transcriptome assembly

The quality of sequencing reads was performed by means of FastQC [2]. Read trimming on quality (Q50) and sequencing adaptors removal was run with Trimmomatic [16], [17].

De novo transcriptome assembly was performed using Trinity [18], using default parameters for the assembly of paired end reads. Mapping and abundance estimation was performed by means of Bowtie [19] using the constructed transcriptome as a reference.
