# Supplementary material for: Data of first de-novo transcriptome assembly of a non-model species, hawksbill sea turtle, Eretmochelys imbricate, nesting of the Colombian Caribean
Source: Data Brief. 2017 Oct 11;15:573–6. doi: 10.1016/j.dib.2017.10.015 (PMC5651488; doi:10.1016/j.dib.2017.10.015)
Supplement: Supplementary file 1 — Supplementary material [file mmc1.pdf]

Conflict of interest

The author declare that they have no competing interests
